# Effect of water-based recovery on blood lactate removal after high-intensity exercise

**DOI:** 10.1371/journal.pone.0184240

**Published:** 2017-09-06

**Authors:** Francesco Lucertini, Marco Gervasi, Giancarlo D'Amen, Davide Sisti, Marco Bruno Luigi Rocchi, Vilberto Stocchi, Piero Benelli

**Affiliations:** 1 Department of Biomolecular Sciences–Division of Exercise and Health Sciences, University of Urbino Carlo Bo, Urbino, Italy; 2 Department of Biomolecular Sciences–Service of Biostatistics, University of Urbino Carlo Bo, Urbino, Italy; Universidad Europea de Madrid, SPAIN

## Abstract

This study assessed the effectiveness of water immersion to the shoulders in enhancing blood lactate removal during active and passive recovery after short-duration high-intensity exercise. Seventeen cyclists underwent active water- and land-based recoveries and passive water and land-based recoveries. The recovery conditions lasted 31 minutes each and started after the identification of each cyclist’s blood lactate accumulation peak, induced by a 30-second all-out sprint on a cycle ergometer. Active recoveries were performed on a cycle ergometer at 70% of the oxygen consumption corresponding to the lactate threshold (the control for the intensity was oxygen consumption), while passive recoveries were performed with subjects at rest and seated on the cycle ergometer. Blood lactate concentration was measured 8 times during each recovery condition and lactate clearance was modeled over a negative exponential function using non-linear regression. Actual active recovery intensity was compared to the target intensity (one sample t-test) and passive recovery intensities were compared between environments (paired sample t-tests). Non-linear regression parameters (coefficients of the exponential decay of lactate; predicted resting lactates; predicted delta decreases in lactate) were compared between environments (linear mixed model analyses for repeated measures) separately for the active and passive recovery modes. Active recovery intensities did not differ significantly from the target oxygen consumption, whereas passive recovery resulted in a slightly lower oxygen consumption when performed while immersed in water rather than on land. The exponential decay of blood lactate was not significantly different in water- or land-based recoveries in either active or passive recovery conditions. In conclusion, water immersion at 29°C would not appear to be an effective practice for improving post-exercise lactate removal in either the active or passive recovery modes.

## Introduction

Short-duration high-intensity exercise leads rapidly to muscular fatigue, which can be defined as the loss of force or power in response to physical exertion resulting in reduced performance [1]. Two of the most important mechanisms involved in exercise-induced fatigue are fibers acidosis and depletion of ATP [2], which lead to large changes in the concentration of metabolites, such as lactate. Lactate is no longer thought to cause fiber acidosis and is believed to provide protection against this process [3]. However its exercise induced rise in the blood coincides with fiber acidosis; hence, it remains a good indirect marker for the onset of fatigue [2, 3]. The rapid removal of lactate following intense exercise remains desirable since it is taken up by both resting muscles and fibers of the same muscle working at lower intensities and used as a carbohydrate fuel source (see [4] for a comprehensive review).

In exercise and sports, blood lactate concentration ([La]_b_) is the most widely used marker of muscular fatigue and several studies have investigated different strategies to enhance lactate removal from the blood after intense physical activity yielding mixing results [5]. Light-to-moderate intensity active recovery has clearly been shown to be a superior lactate clearance strategy compared to passive (resting) recovery [6] after short-duration high-intensity exercise.

Among such recovery strategies, water immersion is the focus of considerable attention among athletes and researchers [7]. From a physical standpoint, water immersion exerts a compressive force on the body, and there is a widely held belief among athletes and trainers that water immersion improves recovery [7]. Indeed, Wilcock *et al*. [8] have hypothesized that hydrostatic pressure, via extracellular fluid transfer to the intravascular compartment and the subsequent increase in cardiac output, may reduce the transport time of the metabolites, including lactate, which accumulate during exercise. However, only two investigations [9, 10] have found enhanced lactate clearance when the recovery from a lactate-accumulating exercise protocol was performed immersed in water rather than on land. Unfortunately, both of these studies were flawed due to their use of heart rate as the control for active recovery intensity since it has been shown that exercises performed at the same oxygen consumption yield significantly higher heart rate responses when they are performed on land compared to when they are performed while immersed at different water depths [11–13]. Therefore, the effect of water on lactate removal from intense exercise needs further investigation.

Accordingly, the aim of this study was to compare the effect of dry-land and water environments on lactate clearance from the blood after short-duration high-intensity exercise. Both active and passive recovery modes were investigated separately.

## Materials and methods

### Participants

Seventeen well-trained young cyclists (see Table 1 for subjects’ characteristics) were recruited. The training level of each cyclist was assessed using a questionnaire [14] (average weekly training hours: 11.3±3.9). The study was approved by the Ethics Committee of the University of Urbino Carlo Bo, and the subjects signed a written informed consent form before being recruited.

**Table 1:** Participants' characteristics and baseline assessments/calculations.

	Age (years)	Height (m)	Weight (kg)	BMI (kg∙m^-2^)	FM (%)	HR_max_ (bpm)	$\dot{V}O_{2\mathrm{peak}}$ (mL∙kg^-1^∙min^-1^)	70% of $\dot{V}O_{2\mathrm{LT}}$ (mL∙kg^-1^∙min^-1^)	[La]_b_ peak_P_-(mmol∙L^-1^)	[La]_b_ peak_A_ (mmol∙L^-1^)
**Mean**	28.4	1.78	71.2	22.4	12.7	191.9	63.2	35.6	13.3	13.3
**SD (±)**	6.4	0.06	5.9	2.1	4.1	8.6	7.7	4.2	2.5	2.3

Average data calculated for seventeen subjects. Abbreviations: BMI, body mass index; FM, fat-mass; HR_max_, maximal heart rate; $\dot{V}O_{2\mathrm{peak}}$, peak oxygen consumption; 70% of $\dot{V}O_{2\mathrm{LT}}$, 70% of the oxygen consumption corresponding to the lactate threshold; [La]_b_ peak, average peak blood lactate concentration achieved before the passive (_P_) and the active (_A_) recovery conditions (average value was computed for twelve subjects for passive recoveries); SD, standard deviation.

### Experimental design

A balanced randomized, crossover study design was used to test the effect of the recovery environment on blood lactate removal. The subjects were scheduled to undergo five experimental sessions at one-week intervals to allow them to fully recover between sessions. For each session, the participants reported to the laboratory well-rested, i.e. without having engaged in strenuous exercise in the previous 48 hours, and at least three hours after a light meal.

In session 1 the cyclists underwent anthropometric and body composition assessments, as well as maximum oxygen consumption ($\dot{V}O_{2}$) and lactate threshold tests (see detailed description of assessments and tests below). Finally, participants underwent a familiarization trial of the peak anaerobic power test (Wingate Anaerobic Test—WAnT).

In sessions 2, 3, 4, and 5, [La]_b_ was measured in four different experimental recovery conditions after the WAnT (see details regarding test and measurement of [La]_b_ below). To raise [La]_b_ rapidly we used the 30-second WAnT protocol, which is commonly used for this purpose [15]. The [La]_b_ peak was identified as the transition from the blood lactate accumulation phase to the clearance phase. In practical terms, [La]_b_ was measured every minute following the completion of the WAnT, and the peak was considered as the [La]_b_ value followed by a measurement equal or lower than that value. The identification of the [La]_b_ peak was temporally out of synchrony with its actual attainment by two minutes because of the time it took for the measuring instrument to yield a result (one minute for each measurement). The actual attainment of the [La]_b_ peak marked the end of the accumulation phase and the beginning of the clearance phase. [La]_b_s during the clearance phase were measured in all four of the following balanced randomly selected experimental conditions: i) passive land-based recovery (PLR)—the subject remained immobile seated on the cycle ergometer used to carry out the WAnT; ii) passive water-based recovery (PWR)—the subject, immersed in water up to his shoulders, remained immobile seated on an underwater cycle ergometer; iii) active land-based recovery (ALR)—the subject peddled on the cycle ergometer used to carry out the WAnT; iv) active water-based recovery (AWR)—the subject, immersed in water up to his shoulders, peddled on an underwater cycle ergometer. Each of these recovery conditions lasted for 31 minutes and was always preceded by two minutes of rest, required for the preparation of the subjects and their proper positioning on the (Aqquactive Bike) underwater cycle ergometer (Aqquatix Ltd., Limena, Italy) under the water-based recovery conditions.

The ambient temperature was monitored constantly during the PLR and ALR recovery conditions. The water temperature during the PWR and AWR was set at 29°C and monitored constantly as well. The water temperature was chosen for two reasons: 1) to replicate a condition that athletes can easily find in real-life settings (29°C is the average temperature set in most swimming pools open to the public); 2) 29°C is the closest water temperature we could get to those of the studies we planned to compare our results with (i.e. 30–31°C in the study of Di Masi et al. [9] and 28–32°C in the study of Ferreira et al. [10]).

The intensity chosen for the ALR and AWR was calculated as a percentage of oxygen consumption at the blood lactate threshold ($\dot{V}O_{2\mathrm{LT}}$), since a recovery calculated using the lactate threshold rather than the maximum $\dot{V}O_{2}$ has been shown to be more suitable [16]. In runners, the intensity that maximizes the clearance of lactate is between 60% and 80% of $\dot{V}O_{2\mathrm{LT}}$ [17, 18], and it has been demonstrated that the kinetic of lactate clearance is not significantly different between runners and cyclists [19–23]. Hence, the intensity of active recovery in the present study was fixed at 70% of $\dot{V}O_{2\mathrm{LT}}$. On a practical level, in both active recovery conditions, oxygen consumption was constantly monitored and the subjects were instructed in real time (every two minutes) to maintain recovery intensity in the range of 65% to 75% of $\dot{V}O_{2\mathrm{LT}}$. The subjects were told to either maintain the resistance of the ergometer and the pedaling frequency or to increase/decrease the resistance of the ergometer (by rotating the brake knob and, if necessary, by changing the pedaling frequency as well) when the intensity fell below or rose above a level that resulted in a ±5% difference between the actual and the target intensity, respectively. Under all the conditions, [La]_b_ was measured at 3 minutes and then every 4 minutes (i.e. 7, 11, 15, 19, 23, 27 and 31 minutes) for a total of 8 blood draws for each recovery condition. [La]_b_ was measured using the same procedure and instrument that was used in the maximum $\dot{V}O_{2}$ test (see detailed description below).

### Assessments

#### Anthropometry and body composition

The body mass index was calculated as the ratio between weight (kg) and height (m^2^), while the percentage of fat mass was calculated by the race-, age-, and sex-specific regression equation of Davidson *et al*. [24], using skinfolds of the biceps, triceps, subscapular and suprailiac as indicated by Durnin & Womerslay [25].

#### Maximum oxygen consumption and lactate threshold

The maximum $\dot{V}O_{2}$ and the lactate threshold were assessed, using the same test, on the SRM cycle ergometer (SRM Italia, Lucca, Italy) with the same settings (height and fore-aft position of the seat, height and distance of the handlebar, and length of the pedal crankarms) and the same type of bicycle pedals for each subject. The original incremental protocol to exhaustion to determine maximum $\dot{V}O_{2}$ calls for a minimum of five and a maximum of nine 4-minute stages, each with resistance increments between a minimum of 20 and a maximum of 50 watts [26] and has been found to be highly reliable for the lactate threshold assessment in cyclists [27]. Although the protocol called for an intensity of 40% of maximum $\dot{V}O_{2}$ for the first stage, we decided to use 30% in light of the fact that to determine the lactate threshold, the blood concentration of lactate at the end of the first stage should not be significantly higher than the resting value [26]. To calculate the resistance to apply to the cycle ergometer corresponding to 30% of maximum $\dot{V}O_{2}$, the theoretical maximum oxygen consumption of each subject was estimated according to Malek *et al*. [28] and then converted into watts (peak) according to the regression equation of Hawley and Noakes [29]. The peak wattage was used to obtain a resistance value equal to 30%, and the difference between the two values was divided over 6 stages. Hence, we were able to determine the necessary watt increase in each stage in order to end the test hypothetically between the 5^th^ and 9^th^ stages (as suggested in [26]). Oxygen consumption was monitored for the duration of the trial (breath-by-breath) using the Cosmed k4b^2^ metabolimeter (COSMED, Rome, Italy), heart rate (HR) was recorded with the Polar RS-800 heart rate monitor (POLAR, Kempele, Finland), and blood lactate was measured (before starting the test and within 30 seconds before the end of each stage) using the Lactate Pro portable blood lactate meter (Arkray, Kioto, Japan) on micro blood samples drawn from the tip of the index finger according to the manufacturer’s instructions. Peak oxygen consumption ($\dot{V}O_{2\mathrm{peak}}$) was identified as the maximum value derived from the 15-breath moving average of oxygen consumption of the entire test, as suggested by Robergs *et al*. [30]. The lactate threshold and the corresponding $\dot{V}O_{2\mathrm{LT}}$ were determined using the algorithm of Bentley *et al*. [31], and implemented using software expressly developed by Newell *et al*. [32], which requires inputting the [La]_b_ and the steady-state oxygen consumption (as the average of the breath-by-breath measurements of the last 30s) for each stage.

#### Wingate Anaerobic Test

The test was conducted on the Peak-Bike cycle ergometer (Monark Exercise AB, Vansbro, Sweden) using a standard protocol [15]. Briefly: i) 12-minute warm up during which the subjects made three short maximum accelerations (for 5 seconds) without friction load at 4, 7 and 10 minutes; ii) 3 minutes of recovery seated on the ergometer; iii) WAnT with friction load equal to 0.098 kp∙kg^-1^ of body weight (i.e. about 10% of body weight).

### Statistical analysis

The following analyses were performed using Excel (Microsoft Office, v.2010) and SPSS Statistics (IBM, v.20) software, with an *α* level of statistical significance of 0.05.

#### Peak blood lactate comparisons

The [La]_b_ peaks yielded before active and passive recoveries were compared using a one-way ANOVA for reaped measures.

#### Recovery intensity comparisons

Subjects' compliance to the target active recovery intensity (i.e., 70% of $\dot{V}O_{2\mathrm{LT}}$) was evaluated. Firstly, the difference between the target recovery intensity and the pooled (ALR and AWR) values of the actual recovery intensity (average percentage of $\dot{V}O_{2\mathrm{LT}}$) was evaluated using a 2-tailed one sample t-test (this comparison was not made for passive recovery). Subsequently, ALR vs. AWR and PLR vs. PWR average values of the actual recovery intensities were compared separately using two 2-tailed, paired sample t-tests. Individual linear regressions were performed for both the active and passive recovery conditions using the actual percentages of $\dot{V}O_{2\mathrm{LT}}$ and the actual percentage of $\dot{V}O_{2\mathrm{peak}}$, respectively, of the whole set of breaths of each subject for the relevant condition. Average slopes and intercepts of ALR and AWR conditions were compared to the expected values of 0 and 70% of $\dot{V}O_{2\mathrm{LT}}$, respectively, by means of two 2-tailed one sample t-tests. Average slopes and intercepts were compared between PLR and PWR using two 2-tailed paired sample t-tests. The same approach as described above was used to compare the HR responses in the active recovery condition (the percentage of HR at the lactate threshold; HR_LT_) to those in the passive recovery condition (the percentage of the maximal HR; HR_max_). All these comparisons were 2-tailed paired sample t-tests since no recovery intensity was planned for HR. All the t-tests were corrected according to Bonferroni’s criterion.

#### Lactate clearance modeling

The lactate clearance kinetics of each recovery condition were modeled on a negative exponential function (as suggested in [33]) whose general form is: y = *a*_0_ + *a*_1_*e*^−*b*x^ where, *a*_0_ is the predicted [La]_b_ at rest, *a*_1_ is the difference between the predicted [La]_b_ peak and *a*_0_ (predicted delta decrease), and *b* is the coefficient of the exponential decay of [La]_b_. Non-linear regression (NLR) fitting was optimized by using the Levenberg-Marquardt algorithm with an initial guess made on the basis of a visual inspection of [La]_b_ over the time plots of each subject, for each recovery condition. A lower limit for predicted [La]_b_ was fixed at *a*_0_ ≥ 0.5 mmol ∙ L^−1^. The coefficient of determination (*R*^2^) was used as a measure of goodness of fit: only regressions resulting in a high *R*^2^ (≥0.8) were considered acceptable and retained for subsequent analyses.

#### Lactate clearance comparisons

Separate linear mixed model analyses for repeated measures were performed for active and passive recovery in order to compare the values of *b*, *a*_0_, and *a*_1_ resulting from the lactate clearance modeling of the land- and the water-based conditions. Active and passive recovery were analyzed separately since it is widely accepted that lactate clearance varies significantly between the two conditions (e.g. see [23]).

## Results

Table 1 shows the results of the anthropometric assessments and parameters measured during the maximum oxygen consumption and lactate threshold test, as well as other parameters that were subsequently calculated. In line with Winter *et al*. [26], in all the tests the wattage increase of each stage always fell within the range of reference, and between 6 and 8 stages were always carried out.

### Peak blood lactates

The [La]_b_ peaks did not differ significantly (*F*_(3,9) =_ 1.528; *p* = 0.273) before starting any recovery condition (see Table 1 and S1 File for raw data of each condition).

### Recovery intensities

Average ambient (PLR and ALR conditions) and water temperature (PWR and AWR conditions) remained constant at 25.3±1.4°C and 29±0.5°C, respectively. Five participants did not undergo the passive recovery conditions; therefore, the number of subjects for PLR and PWR decreased to twelve.

The α level of significance corrected according to the Bonferroni's criterion resulted in a *p* level of statistical significance of 0.017. Pooled ALR and AWR average recovery intensity did not differ significantly from the target recovery intensity of 70% of $\dot{V}O_{2\mathrm{LT}}$ (*p* = 0.639). Average values for passive recoveries were computed for nine subjects due to technical problems we encountered in sampling oxygen consumption in three subjects. Actual average oxygen consumptions during active recovery did not differ significantly between land and water conditions (*p* = 0.381), and the linear regression parameters intercept and slope of both environmental conditions did not differ significantly from 70 (land: *p* = 0.249; water: *p* = 0.899) and 0 (land: *p* = 0.147; water: *p* = 0.193), respectively. Regarding HR during active recovery, average values (*p* = 0.004) and regression intercepts (*p* = 0.016) differed significantly between the environments, while regression slopes did not (*p* = 0.306). Average values of oxygen consumption during passive recovery were significantly different (*p* = 0.006) in the environments, whereas the regression parameters intercept (*p* = 0.069) and slope (*p* = 0.466) were not. HR average values (*p* = 0.007) and regression slopes (*p* = 0.002) of passive recovery differed significantly between the environments, while regression intercepts did not (*p* = 0.581). See Table 2 for details regarding the comparisons of the recovery intensities for both active and passive conditions.

**Table 2:** Comparisons of the recovery intensity between land- and water-based clearance conditions for both active and passive recovery modes.

			Active recovery		Passive recovery	
			$\dot{V}O_{2\mathrm{LT}}$	HR_LT_	$\dot{V}O_{2\mathrm{peak}}$	HR_max_
**Actual intensities**						
	**Land**					
		**Mean % (±SD)**	70.1 (3.9)	83.8 (8.9) *	12.6 (3.1) *	47.4 (3.6) *
	**Water**					
		**Mean % (±SD)**	69 (3)	77.5 (6.4) *	13.9 (3.2) *	43.1 (3.9) *
**Linear regressions**						
	**Land**					
		**Intercept (±SD)**	71.1 (3.8)	83.2 (8.5)	15.1 (4.2)	50.3 (3.9)
		**Slope (±SD)**	-0.0 (0.0)	0.0 (0.0)	-0.0 (0.0)	-0.0 (0.0) *
	**Water**					
		**Intercept (±SD)**	70.2 (5.1)	77.9 (6.7) *	16. (4.3)	49.6 (3.9)
		**Slope (±SD)**	-0.0 (0.0)	-0.0 (0.0)	-0.0 (0.0)	-0.0 (0.0) *

Average data were calculated for seventeen and nine subjects for active and passive recovery, respectively. Linear regression parameters were calculated for sixteen and eleven subjects for active and passive recovery, respectively. Abbreviations: $\dot{V}O_{2\mathrm{LT}}$, oxygen consumption at the lactate threshold; HR_LT_, heart rate at the lactate threshold; $\dot{V}O_{2\mathrm{peak}}$, peak oxygen consumption; HR_max_, maximal heart rate; SD, standard deviation;

*, significantly different from the other recovery environment.

### Lactate clearance modeling and comparisons

NLRs were not acceptable (i.e. *R*^2^<0.5 or non-plausible predicted values) in one subject for water-based recovery and in one subject for land recovery; therefore, lactate clearance comparisons were performed on sixteen and eleven subjects for active and passive recovery, respectively. The average goodness of fit for the NLRs of each condition was very high (ALR, *R*^2^: 0.98; AWR, *R*^2^: 0.99; PLR, *R*^2^: 0.97; PWR, *R*^2^: 0.96).

**Fig 1:**
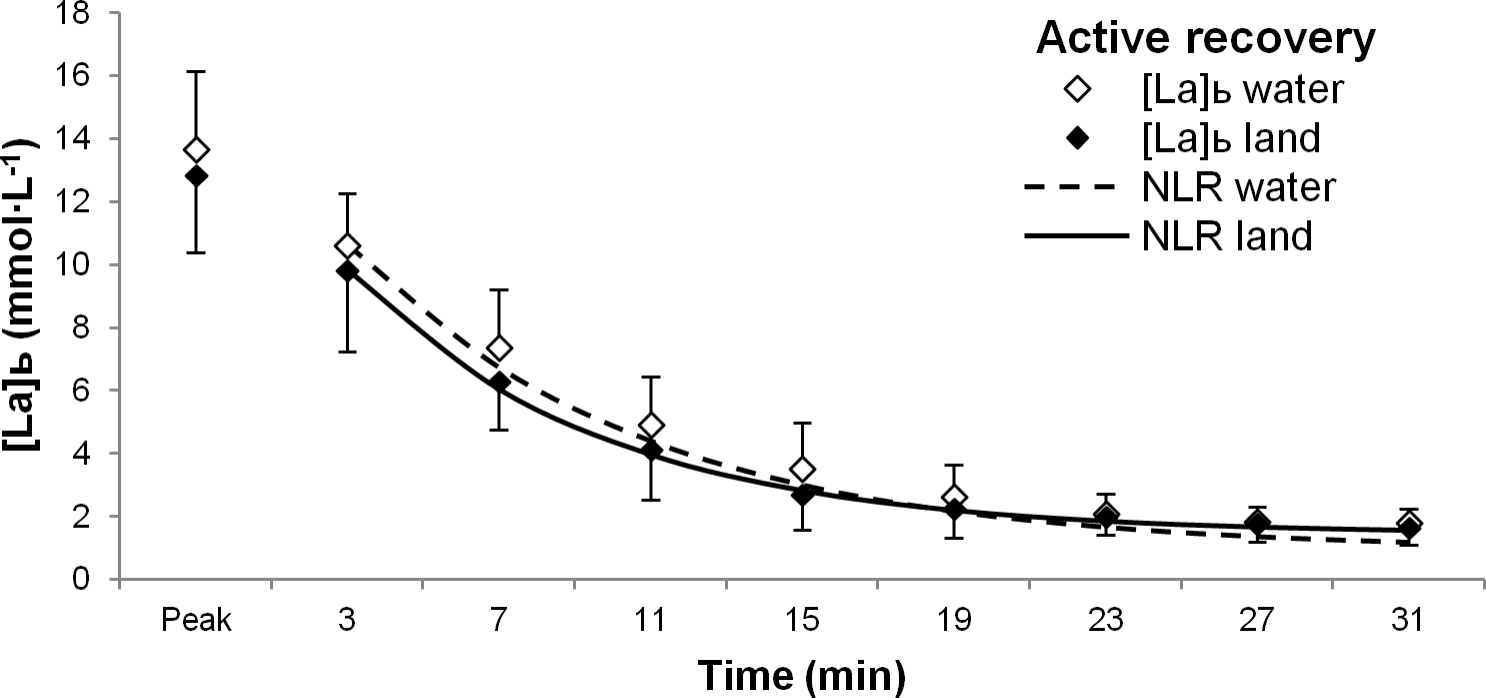
Blood lactate removal during active recovery. Blood lactate concentration decays during water-based (white diamonds) and land-based (black diamonds) active recovery conditions following a 30-second all-out bout of cycling. Non-linear regression curves are also shown for water-based (dashed line) and land-based (solid line) recovery. Abbreviations: [La]_b_, blood lactate concentration; NLR, non-linear regression.

**Fig 2:**
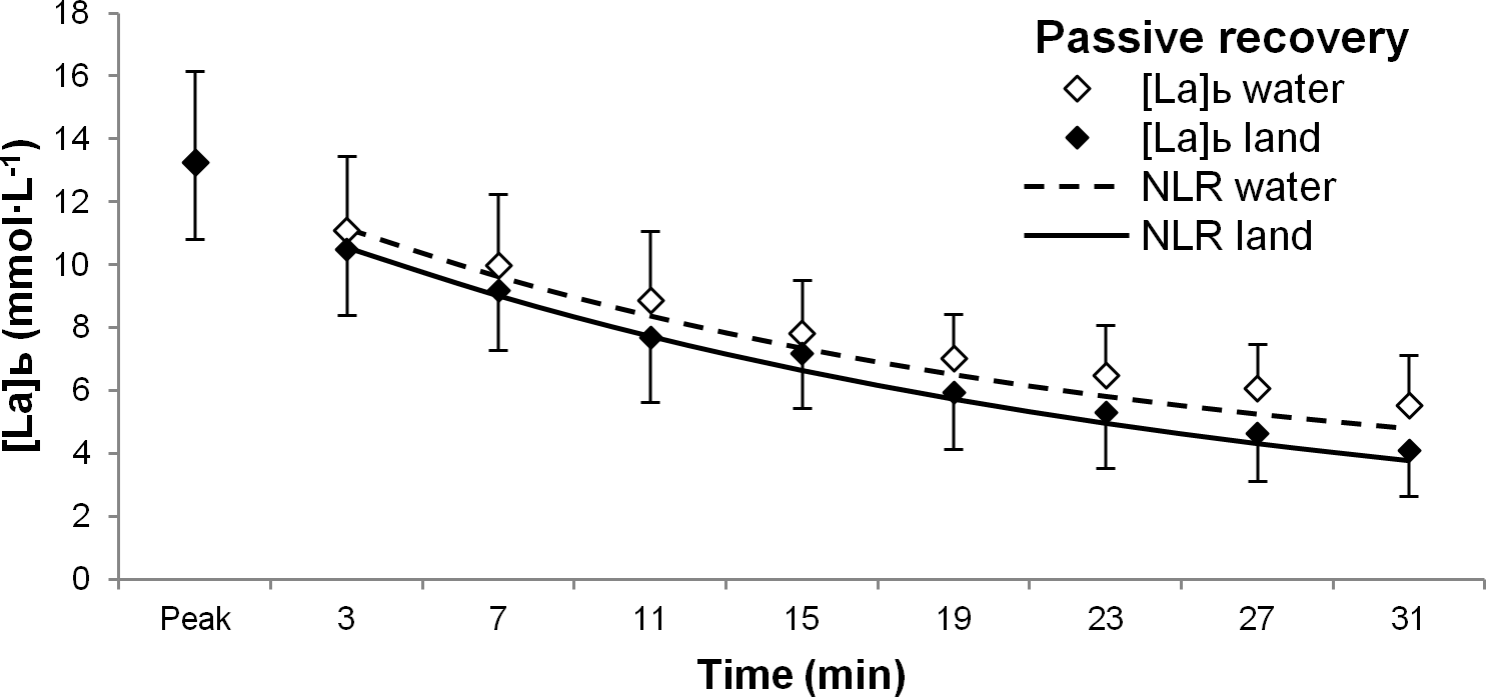
Blood lactate removal during passive recovery. Blood lactate concentration decays during water-based (white diamonds) and land-based (black diamonds) passive recovery conditions following a 30-second all-out bout of cycling. Non-linear regression curves are also shown for water-based (dashed line) and land-based (solid line) recovery. Abbreviations: [La]_b_, blood lactate concentration; NLR, non-linear regression.

The environment of recovery did not affect any parameter of the negative exponential equations (*b*, *a*_0_, *a*_1_) in either active (Fig 1; *F*_(1,16)_ = 0.372, *p* = 0.551; Cohen’s D effect size: *b* = 0.49; *a*_0_ = 0.64; *a*_1_ = 0.36) or passive (Fig 2; *F*_(1,11)_ = 1.387, *p* = 0.264; Cohen’s D effect size: *b* = 0.24; *a*_0_ = 0.84; *a*_1_ = 0.5) recovery conditions.

## Discussion and conclusions

The results of the present investigation clearly show that immersion to the shoulder in 29°C water does not improve lactate clearance in active or passive recovery. These results are not in agreement with the only two studies on this specific topic in literature, which showed an overall positive effect of the water environment in the clearance of blood lactate [9, 10]. These conflicting results can probably be attributed to the substantial differences in the experimental design of the studies, in particular, the modalities of intensity control in active recovery and the identification of the starting point of the recovery phase.

In the study by Di Masi *et al*. [9], the subjects recovered actively in both land-based and water-based conditions at an intensity equal to 65% of the age-predicted HR (calculated with the 220-age formula), whereas in the study by Ferreira *et al*. [10], the subjects recovered actively in land-based and water-based conditions pedaling at 85% of the HR corresponding to the ventilatory threshold. Hence, both studies used HR to equalize the intensity levels of active recovery in the two different environmental conditions, even though it is known that during submaximal exercise, oxygen consumption being equal, HR in water is lower than it is on land by approximately 10–15 bpm [11, 13, 34–36]. Although both Di Masi *et al*. [9] and Ferreira *et al*. [10] correctly chose percentages of HR that theoretically determine workloads under the lactate threshold [16], the use of HR implies that subjects in both investigations made an active recovery that varied in terms of metabolic intensity according the conditions (land-based or water-based). Hence, the measured [La]_b_ levels could not be compared because the intensity of the active recovery has a considerable effect on blood lactate clearance capacity [18]. On the other hand, in the present study, the intensity of the active recovery was monitored in both environmental conditions on the basis of the true oxygen consumption, which guarantees the comparability of intensity in the various experimental conditions. Moreover, we had the subjects recover actively at an intensity that was undoubtedly lower than the lactate threshold (70% of $\dot{V}O_{2\mathrm{LT}}$), under continuous monitoring by members of our research team, who informed the participants every time intensity fell below or exceeded the target level. The absence of a statistically significant difference in the intensities of oxygen consumption between the environments of recovery in the active condition points to the suitability of our experimental design. On the contrary, HR comparisons revealed a significantly lower average active recovery intensity (about −6%) in water compared to land conditions, which is in line with the above-mentioned studies and further supports our choice of using oxygen consumption as the control for recovery intensity. Unfortunately, passive recovery average values of oxygen consumption were found to be slightly (about +1.3% of $\dot{V}O_{2\mathrm{peak}}$) but significantly higher in water than on land. This result is in line with other studies (e.g. see Park *et al*. [34]) carried out under similar conditions of water temperature and resting duration while seated on the cycle ergometer. Under those water-based conditions, the average skin temperature reduces significantly [34] and oxygen consumption and metabolism increase to maintain core temperature [8]. In support of this view, five participants did not well tolerate the water environment during passive recovery and after about 20 minutes started to feel too cold to conclude the 31-minute recovery and voluntarily interrupted the experiment. However, since both the regression intercept and slope of passive recovery oxygen consumption did not differ significantly between the environments, we are confident that comparisons of passive recovery lactate clearance between land and water-based conditions can still be drawn. That would not have been the case if HR had been used as the control for passive recovery intensities since average values were found to be significantly higher (about +4.3% of HR_max_) on land than in water and regression slopes differed significantly between the environments.

The experimental protocol of this investigation called for a high number of blood samples during the recovery phase (8 blood draws in 31 minutes). This allowed us to model the [La]_b_ decay over time and to make more accurate comparisons between the two environmental conditions compared to previous investigations [9, 10], hence obtaining a more complete and detailed picture of the kinetics of lactate clearance.

In addition, the clear identification of the onset of the blood lactate clearance phase is indispensable to correctly assess its kinetics. In the studies by Di Masi *et al*. [9] and Ferreira *et al*. [10], a comparison was drawn among the [La]_b_ values obtained from blood draws made starting at a time chosen arbitrarily after the completion of the trial used for [La]_b_ accumulation. In particular, in the investigation of Di Masi *et al*. [9], the onset of the recovery phase was established one minute after the termination of the anaerobic exercise, whereas in the Ferreira *et al*. protocol [10], the onset of the recovery phase was established at five minutes after the termination of the exercise. It has been shown that following a short bout of maximal physical exertion, the moment of peak blood lactate is rather unpredictable [33, 37]. Hence, considering the low frequency of the blood draws in these investigations, values that actually belong to the lactate accumulation phase immediately following the end of the maximal exercise test, may have been included among the values used to establish the average rate of blood lactate clearance identified by Di Masi *et al*. [9] and Ferreira *et al*. [10] for the two environmental conditions. This may account for the differences between the results of the above-mentioned investigations and the results obtained in the present study, which only used [La]_b_ values that clearly belonged to the clearance phase after the maximal trial because the experimental design called for the identification of the peak [La]_b_. In line with the interindividual variability regarding the time taken to reach [La]_b_ peak values reported in literature [33, 37], in the present study the [La]_b_ peak was recorded on average after 3.6±1.2 minutes from the end of the WAnT, despite the [La]_b_ peaks were not significantly different before starting any of the recovery conditions.

The main limitation of our investigation lies in the delay between the end of the WAnT and the identification of the [La]_b_ peak and between the identification of the peak and the beginning of the selected recovery condition. In the first case, the limitation is due to the lactate meter used in this study, which takes one minute to yield a measurement; hence, the [La]_b_ peak was always identified after two minutes from the onset of the clearance phase. In the second case, we elected to delay the start of all the recovery conditions by two minutes to allow for the correct positioning of the subjects on the cycle in the water-based recovery conditions. Hence, the [La]_b_ of the first four minutes of recovery following the peak were never recorded. Nevertheless, since the kinetic of lactate clearance follows a well-known negative exponential pattern [33], it can be hypothesized that the pattern of lactate clearance from the fourth minute onwards is representative of the kinetics of the first four minutes. In any case, the aim of this investigation was to assess clearance differences attributable to the various conditions. Therefore, on a practical level, the short delay more accurately reflects the reality of recovery for athletes, who after a maximal exertion take a few minutes before starting their recovery immersed in water.

In conclusion, our main finding suggests that, contrary to what has been postulated in literature to date, the water environment does not produce different effects than the land environment on the kinetics of blood lactate clearance during active recovery from intense exercise. Hence, immersion in 29°C water would not appear to be a practice that can speed up post-exercise lactate removal in either the active or passive mode, and is therefore not advisable under the specific conditions investigated in the present study.

Additional research on this topic is needed and future studies should take into account possible variations in aquatic environmental conditions (water temperature, exercise type and mode, depth of immersion, etc.) to further investigate the potential of the water environment in modulating blood lactate clearance after intense exercise.

## Supporting information

S1 File Raw data. (XLSX)
